# Biomechanical Evaluation of Novel Tendon Coaptation Technique: A Potential Application for Tendon Transfers

**DOI:** 10.1016/j.jhsg.2024.11.007

**Published:** 2024-12-10

**Authors:** Devin W. Collins, Miguel A. Diaz, Nino Coutelle, Mariel McLaughlin, Damir Pamic, Peter Simon, Michael C. Doarn

**Affiliations:** ∗ Florida Orthopaedic Institute, Tampa, FL; † Department of Orthopaedics & Sports Medicine, University of South Florida, Tampa, FL; ‡ Foundation for Orthopaedic Research & Education, Tampa, FL; § Department of Plastic and Reconstructive Surgery, University of South Florida, Tampa, FL

**Keywords:** CoNextions, Pulvertaft, Tendon transfer

## Abstract

**Purpose:**

The purpose of this study was to evaluate the biomechanical performance and time to completion of the Pulvertaft weave technique, the CoNextions tendon repair system only, and a combination of single Pulvertaft weave with the CoNextions repair.

**Methods:**

A total of 22 cadaveric hands were dissected, and the extensor pollicis longus tendons were harvested and divided into the following three groups: (1) Pulvertaft weave, (2) Pulvertaft weave + CoNextions, and (3) CoNextions. Each sample underwent axial loading in a cyclical fashion, followed by ramp to failure. Metrics of interest were suture time, displacement, stiffness, ultimate failure load, and failure mechanism were recorded.

**Results:**

Time required to instrument each configuration was significantly different across all groups; CoNextions group was the fastest. For cyclic loading, no significant differences in elongation were detected between the groups. The displacement at ultimate failure load, on average, for the Pulvertaft (10.6 mm) and the Pulvertaft and CoNextions combination (10.2 mm) were not significantly different compared with CoNextions (6.5 mm). The stiffness during cyclic loading was similar between the Pulvertaft (38.8 N/mm) and the Pulvertaft and CoNextions combination (36.7 N/mm), and both were found to be significantly stiffer compared with the CoNextions (28.0 N/mm). During the ramp to failure, no significant differences in stiffness were detected. The Pulvertaft had the largest ultimate failure load at 67.5 N.

**Conclusions:**

The Pulvertaft weave and CoNextions tendon repair device demonstrated similar performance to traditional Pulvertaft tendon weave. The combination technique is faster than the traditional tendon weave and displayed improved performance compared with CoNextions repair alone.

**Clinical relevance:**

The combined use of novel tendon repair with a single tendon weave allows for faster tendon coaptation and equivalent strength as a Pulvertaft weave.

Hand injuries account for somewhere between 14% and 27% of injuries that present to the emergency department annually.^1^, ^2^, ^3^, ^4^ Tendon injuries are one of these and can occur in various ways. Trauma to nerves is another way that tendon dysfunction can develop. These injuries can result in loss of mobility, abnormal joint kinematics, and alterations to adjacent tissues. Tendon transfers are commonly employed throughout the body to regain function after tendon ruptures and nerve injuries. The primary goal of tendon transfers was to restore function across joints in both the upper and lower extremities. The ideal tendon transfer allows for matched muscle strength, tendon excursion, and appropriate tensioning of the transfer.^5^ When considering tendon transfers, it is critical to understand the expendability of the donor muscle–tendon unit and loss of muscle strength to the recipient tendon.^6^

First described by Pulvertaft in 1956, the Pulvertaft weave has been proven effective for tendon transfers and has shown to be a reliable technique.^7^, ^8^, ^9^ There have been various biomechanical studies comparing the strength of Pulvertaft weave with side-to-side tendon suture, Pulvertaft modification, and novel tendon techniques.^10^, ^11^, ^12^, ^13^, ^14^, ^15^, ^16^, ^17^, ^18^, ^19^, ^20^ When compared with Pulvertaft, multiple studies demonstrated that side-to-side repair had a higher force to failure than the Pulvertaft technique without any difference in bulk.^10^^,^^11^^,^^18^ Although additional techniques have proven stronger than Pulvertaft, they have resulted in an even bulkier reconstruction, which is particularly unfavorable in tendon reconstruction.^13^^,^^17^ Despite advancements in materials and surgical technique, postoperative complications including tendon repair rupture and adhesion formation remain common.

Of these recent advances, the CoNextions tendon repair system (CoNextions Inc) has gained popularity because of its strength, ability to allow active motion immediately, and decreased adhesion formation.^21^, ^22^, ^23^ This tendon repair system may be applicable to tendon transfers and allow faster recovery and decreased rehabilitation time among individuals undergoing transfers. However, there is currently no data on the biomechanical performance of this system compared with coaptation techniques used for tendon transfers.

The purpose of this study was to evaluate the biomechanical performance (elongation, stiffness, and ultimate failure) and time to completion of the Pulvertaft weave technique and compare it with the performance of the CoNextions tendon repair system alone and a combination of a single Pulvertaft weave with the CoNextions tendon repair device. We hypothesize that a single Pulvertaft weave with the CoNextions tendon repair device will have a faster tendon transfer time when compared with a traditional Pulvertaft weave and have similar biomechanical performance. Additionally, it was hypothesized that the combination technique will have improved performance compared with CoNextions end-to-end coaptation alone for tendon transfers.

## Methods

### Dissection and sample preparation

**Figure 1:**
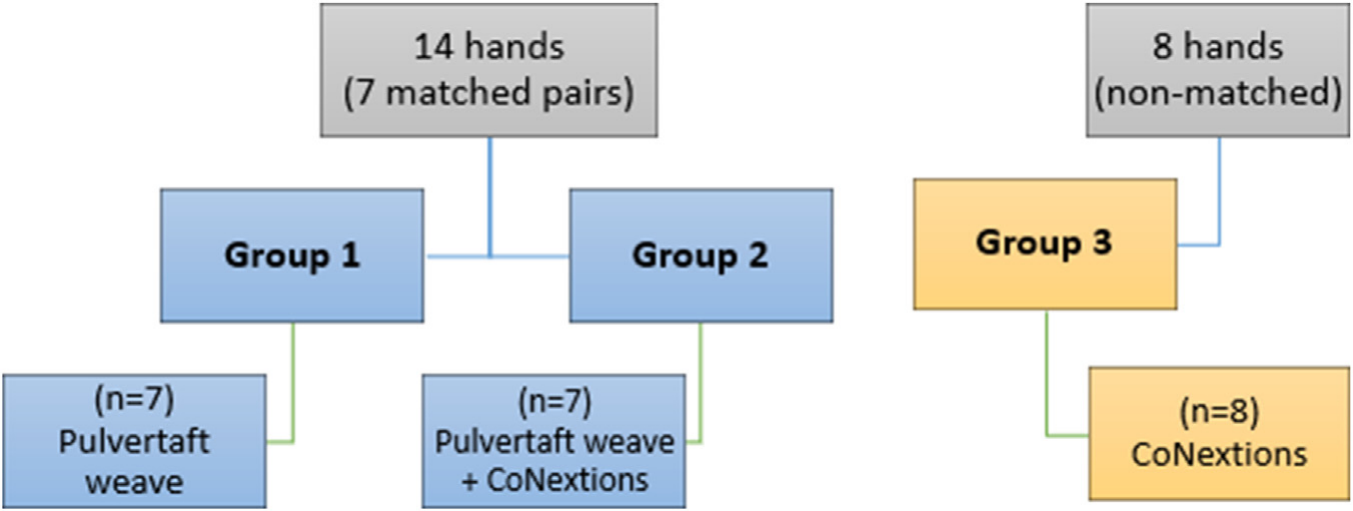
Test grouping.

A total of 22 fresh frozen cadaveric extensor pollicis longus (EPL) tendons were harvested and divided into the following three groups: (1) Pulvertaft weave, (2) Pulvertaft weave + CoNextions, and (3) CoNextions. Groups 1 and 2 consisted of seven matched pairs each (four women; three men; average age: 75 years [range: 58–93 years]), whereas group 3 (four women; four men; average age: 81 years [range: 68–99 year]) had eight nonmatched tendons (Fig. 1). All tendons were harvested by dissecting the EPL tendons from each specimen and obtaining the entire length of the tendon up to the musculotendinous junction. The EPL tendon was chosen due to ease of harvest with long tendon length and to model a common tendon transfer in the upper extremity of extensor indicis proprius to extensor pollicis longus. Care was taken not to damage the tendon during harvest. All tendon transfer techniques were performed by a single fellowship-trained board-certified hand surgeon experienced in the Pulvertaft weave technique and the CoNextions tendon repair device system technique. The time required to perform each repair per group was recorded.

**Figure 2:**
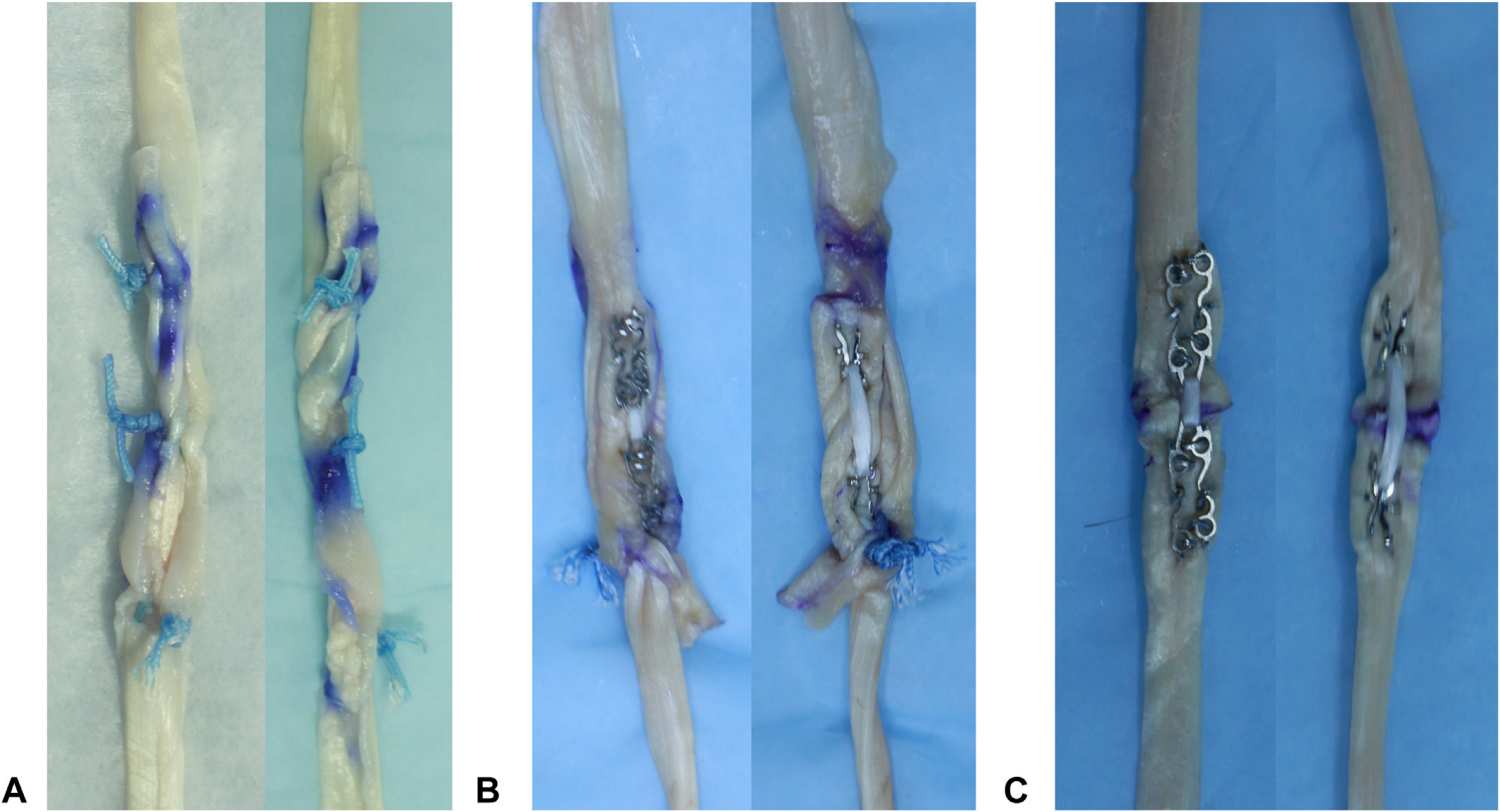
Illustration of tendon repairs where (**A**) is Pulvertaft Weave only, (**B**) is Pulvertaft weave + CoNextions, and (**C**) is CoNextions-only.

Group 1 was instrumented with only the Pulvertaft weave. A total of three weaves were performed. A tendon passer was used to weave the primary tendon through the secondary tendon. At each weave, a horizontal mattress suture was used to secure the weave. A 3–0 FiberWire suture (Arthrex) was used for this. The end of the primary tendon was laid flat on the secondary tendon after three weaves and sutured to each other with a through-and-through horizontal mattress suture (Fig. 2A).

Group 2 was instrumented with Pulvertaft weave and supplemented with the use of the CoNextions tendon repair system. A single Pulvertaft weave was performed with a tendon passer. The weave was secured with one horizontal mattress suture with 3–0 FiberWire.

The primary tendon end was laid on top of the secondary tendon, and the CoNextions device was placed with both tendons compressed between the system and secured in place (Fig. 2B).

Group 3 was instrumented with the CoNextions repair system as a standalone technique of securing the two tendon ends together. The primary and secondary tendons were brought together in an end-to-end fashion with a 3–0 Vicryl stay suture to approximate the tendons. The CoNextions device was then applied as per the standard recommended technique (Fig. 2C).

In a comparison of the three groups, the Pulvertaft weave was the bulkiest followed by the weave + CoNextions. The CoNextions alone was the least bulky.

### Biomechanical testing

**Figure 3:**
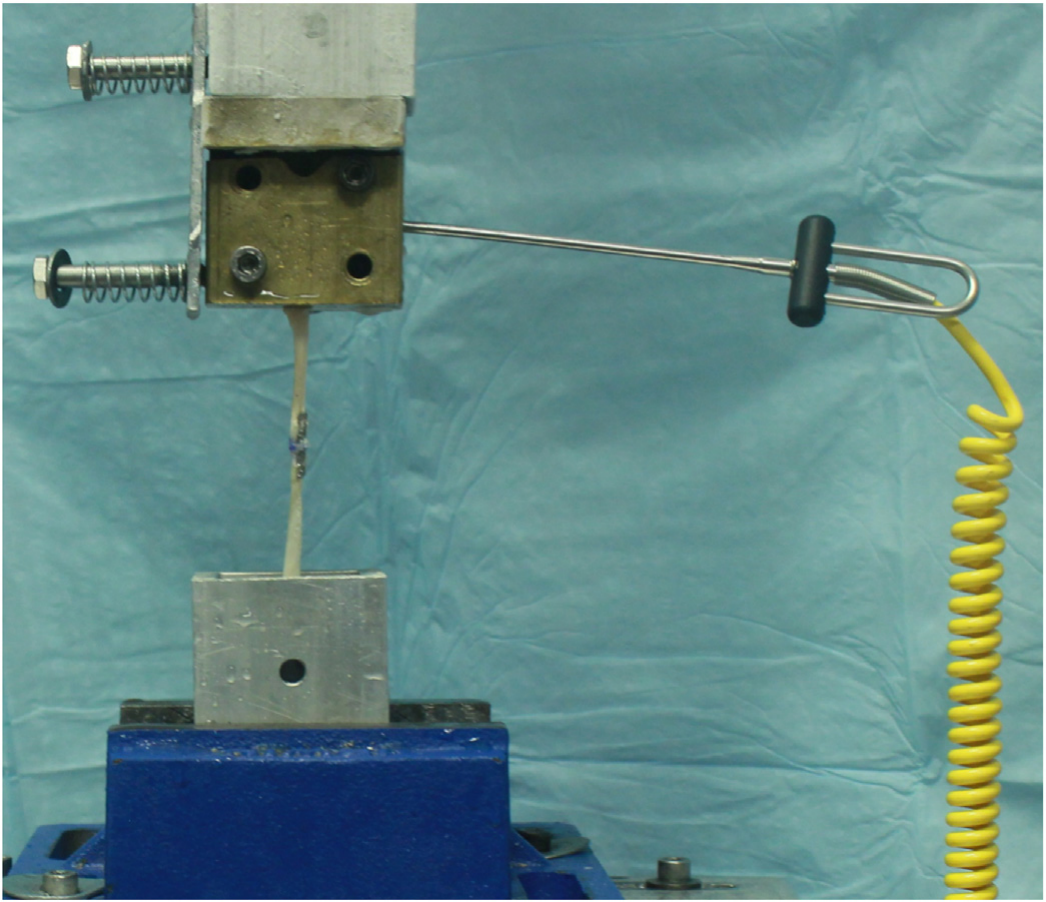
Biomechanical testing setup with mounted sample in Cyroclamp with temperature probe.

Testing was conducted with a servohydraulic MTS Bionix test frame (MTS Systems). Specimens were attached to the MTS actuator by the tendon through a cryoclamp cooled to a temperature of −22°C, a critical temperature that has been previously used to ensure secure coupling between the tendon and the clamp.^24^ The proximal and distal ends of the tendons were both clamped to the cryoclamp and ensured that all tendons were tested under the same conditions. Tensile forces were applied in line with the tendon (Fig. 3). Our mechanical testing protocol was based on previously published models,^8^^,^^14^^,^^19^^,^^25^, ^26^, ^27^, ^28^ with slight modifications. All constructs were preconditioned to normalize viscoelastic effects and testing variability through the application of static load and cycle testing. For stress relaxation, a load of 5 N was applied and held for 1 minute. The displacement was recorded and then zeroed. From this starting point, cyclic loading (in load control) from 5 to 25 N at 0.5 Hz for 100 cycles was performed. After cyclic loading, the samples were ramped to failure testing at 40 mm/min. During cyclic loading, displacement data were collected from the actuator’s linear variable differential transformer at 1, 10, 20, 40, 60, 80, and 100 cycles as a measure of progressive construct elongation (mm). Elongation during cyclic loading was presented as the average displacement of the last 10 cycles. During failure testing, stiffness (N/mm), ultimate failure load (N), and failure mechanisms were recorded. Failure was qualified as the first significant decrease in the monotonically increasing force profile, whereas stiffness was defined as the linear portion of the force–displacement curve. Failure mode was classified and assessed visually as either a suture pull-out, suture failure, or breakage of the tendon itself. The ultimate failure load was determined by the peak value on the load–displacement curve, whereas the stiffness was the slope of the best-fit line on the ascending linear region of the load–displacement curve.

### Statistical analysis

Based on data from the literature, a priori power analysis was performed with an effect size (Cohen’s d) of 1.4 to compute the required sample size to detect a difference between the matched paired groups (G∗Power 3.1.9.2, Franz Faul).^25^^,^^28^ Assuming a power of 0.8 and a type I error rate of 0.05, a total sample size of 9 (three samples per group) powered the study to 0.83. The choice of 22 total samples powered the study above 0.95, allowing for any possible tissue rejection or unforeseen failures while retaining proper study power. A one-way analysis of variance was used to compare time, displacement, stiffness, and failure load across the three groups. This was followed by Tukey’s HSD as post hoc test. Commercially available software was used for all comparisons at a significance threshold of 0.05. Data were presented as mean ± standard deviation.

## Results

### Demographics

No differences were observed between the testing groups for age (*P* = .357), weight (*P* = .949), or body mass index (*P* = .297). Similarly, no differences were observed between the groups and tendon dimensions (length, thickness, and width). The average length for all tendons was 14.4 ± 1.2 mm, thickness was 1.8 ± 0.4 mm, and widths were 4.4 ± 0.9 mm.

### Time

The time required to instrument each configuration was significantly different across all groups (*P* < .001), where the CoNextions group was the fastest taking on average 71 ± 22 seconds. The next fastest group was the combination of CoNextions and Pulvertaft weave at an average of 162 ± 34 seconds, and finally, the Pulvertaft weave at 303 ± 20 seconds.

### Displacement

During the cyclic loading, no significant differences in elongation were detected between the various techniques where the Pulvertaft weave was 0.28 ± 0.01 mm, CoNextions was 0.30 ± 0.02 mm, and the Pulvertaft and CoNextions combination was 0.29 ± 0.02 mm (*P* = .56). The displacement at ultimate failure load, on average, for the Pulvertaft (10.6 ± 3.7 mm) and the Pulvertaft and CoNextions combination (10.2 ± 3.0 mm) was not statistically different when compared with the CoNextions (6.5 ± 0.5 mm; *P* = .053).

### Stiffness

The stiffness during cyclic loading was similar between the Pulvertaft (38.8 ± 5.7 N/mm) and the Pulvertaft and CoNextions combination (36.7 ± 6.1 N/mm, *P* = .79). Both configurations were found to be significantly stiffer compared with the CoNextions-only, 28.0 ± 6.4 N/mm (Pulvertaft: *P* = .013; Combination: *P* = .047). However, during the ramp to failure, no significant differences in stiffness were detected between the various techniques where the Pulvertaft weave was 26.4 ± 5.6 N/mm, CoNextions was 22.8 ± 6.7 N/mm, and the Pulvertaft and CoNextions combination was 26.8 ± 5.2 N/mm (*P* = .43).

## Failure load and failure model

The Pulvertaft had the largest ultimate failure load at 67.5 ± 19.4 N and significantly larger than the Pulvertaft and CoNextions combination 49.0 ± 7.2 N (*P* = .032) and the CoNextions-only, which had an ultimate failure load of 31.9 ± 3.6 N (*P* = .00023). Moreover, the Pulvertaft and CoNextions combination had a significantly larger ultimate failure load compared with CoNextions-only (*P* = .0034).

**Figure 4:**
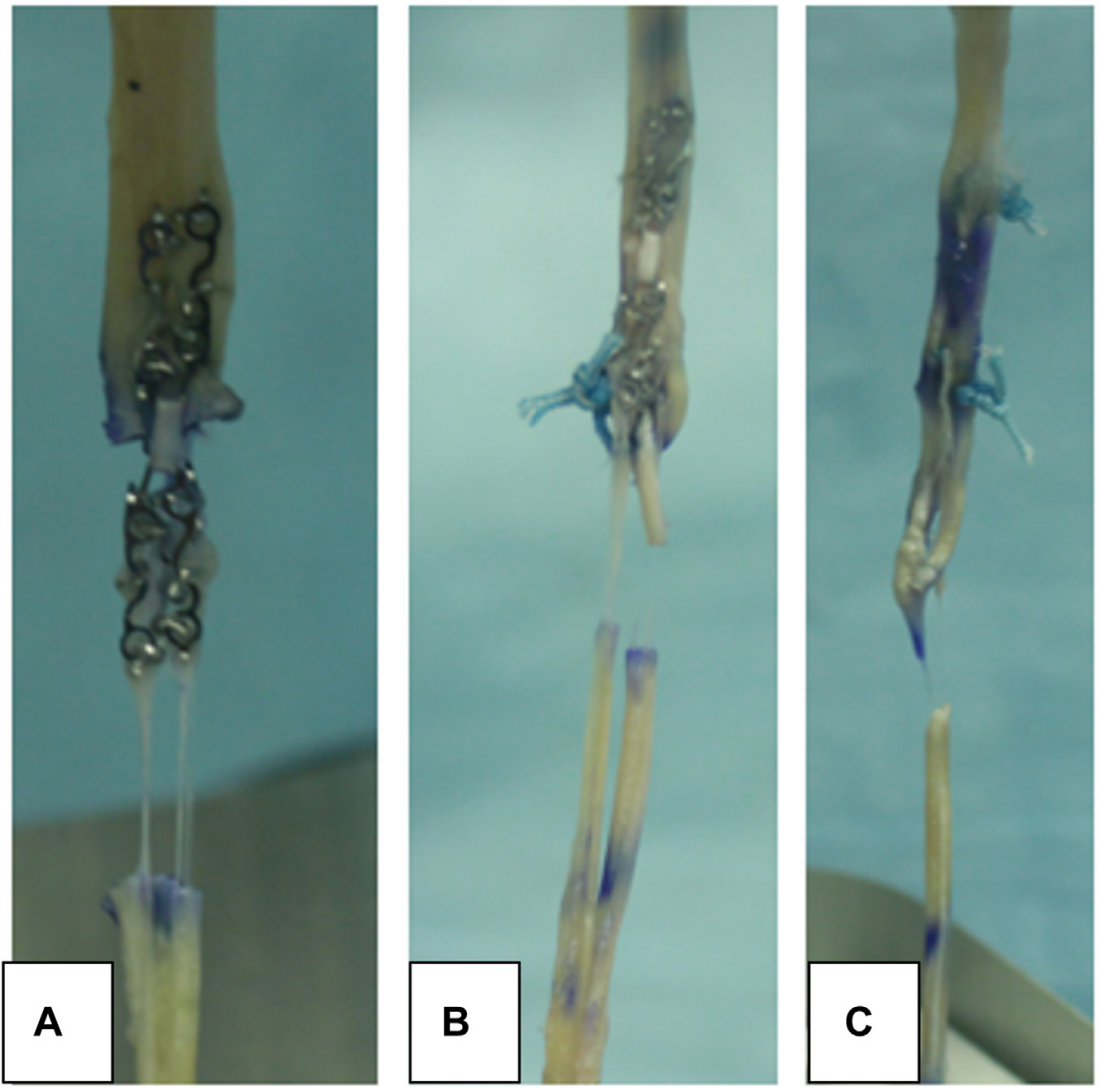
Example of the two failure modes observed where (**A**) CoNextions-only experienced failure by tendon tear-through, where the suture/metal had a “cheese-wire” effect cutting through the tendon. The second mode of failure was described as failure at the first knot followed by tendon tear-through as shown by (**B**) Pulvertaft and CoNextions combination and (**C**) Pulvertaft-only.

Two failure modes were observed during the ramp to failure testing (Fig. 4). The first was described as a tendon tear-through, where the suture/metal had a “cheese-wire” effect cutting through the tendon. All the CoNextions-only groups failed in this manner. The second mode of failure was described as failure at the first knot followed by tendon tear-through. All the Pulvertaft-only and Pulvertaft and CoNextions combinations failed in this fashion, where the failure initiated at or below the first knot and progressed into tendon tear-through.

Overall, every sample in the Pulvertaft and the Pulvertaft and CoNextions combination groups survived the preconditioning test cycles. For the CoNextions-only group, 75% (6/8) survived the preconditioning test. The 25% (2/8) that failed, did so halfway through testing with a load of 19 ± 1.0 N.

## Discussion

The purpose of this study was to evaluate the biomechanical performance (elongation, stiffness, and ultimate failure) and time to completion of the Pulvertaft weave technique and compare it with the performance of the CoNextions tendon repair system alone and a combination of a single Pulvertaft weave with the CoNextions tendon repair device. We hypothesize that a single Pulvertaft weave with the CoNextions tendon repair device will have a faster repair time when compared with a traditional Pulvertaft weave and have similar biomechanical performance. Additionally, it was hypothesized that the combination technique will have improved performance compared with CoNextions end-to-end repair alone for tendon transfers.

Two failure modes were observed during the ramp to failure testing. The first was described as tendon tear-through. It was observed here that the suture/metal had a “cheese-wire” effect cutting through the tendon. The CoNextions-only group failed in this manner, and the device itself did not break. This is likely due to the stiffness of the construct itself compared with the tendon tissue. The second mode of failure was described as a failure at the first suture knot followed by tendon tear-through. All the Pulvertaft-only and Pulvertaft and CoNextions combinations failed in this fashion, where the failure initiated at or below the first knot and progressed into tendon tear-through. This is consistent with what has been reported in the literature as well as seen in personal clinical experience.^14^^,^^16^^,^^19^^,^^25^

Our hypothesis was confirmed that a single Pulvertaft tendon weave with the addition of one CoNextions device is biomechanically as strong as the traditional three weave Pulvertaft technique. Testing revealed that the stiffness and load to ultimate failure were both similar between the weave and weave plus CoNextions groups. Our results show that this combination technique is significantly faster than the three weave. For the CoNextions-only group, only 75% survived the preconditioning test and the 25% that failed, did so halfway through testing with a relatively low load. In addition, this technique had the lowest stiffness and load to failure and had the largest displacement at ultimate failure.

The minimum strength threshold a tendon transfer should withstand is unclear. Reported values of the ultimate failure load for tendon transfer reconstructions vary greatly in literature and are highly dependent on the type (flexor vs extensor) and quality of tendon (human vs animal).^6^^,^^10^^,^^13^, ^14^, ^15^^,^^18^^,^^19^^,^^27^^,^^29^^,^^30^ Koopman et al^18^ assessed flexor and extensor tendon transfer reconstructions with Pulvertaft weave and found that the mean load to failure in the flexor tendon was 87 and 59 N in the extensor tendon. It has been reported that the functional after-care tendon repair strength should be between 9 N for passive mobilization and 35 N for active mobilization for the finger, but others have reported higher threshold values between 75 and 120 N for upper-extremity tendon transfers.^8^^,^^13^^,^^29^, ^30^, ^31^, ^32^ Given our tendon samples were EPL, our values for failure load do not meet the upper threshold values likely regarding the flexor tendon; however, two groups did meet or surpass the values reported for extensor tendons. One group had a mean ultimate failure load below 35 N. Based on these results, it would not be recommended to do end-to-end tendon transfer with the CoNextions device alone.

Clinically, the difference in tendon transfer time could be beneficial with less operating room time and time under anesthesia, especially if multiple tendon transfers and/or other concomitant procedures are being performed. Furthermore, the results shown here should allow for a similar return to motion, possibly earlier with the potential for less scarring/adhesions after tendon transfer due to less tendon trauma from suturing and from tendon passage. This could give less tissue bulk in the transfer. Although bulk was not measured in this study, bulkiness of the Pulvertaft weave has been investigated, as it has been postulated that the bulk of the transfer can lead to increased friction and thus more adhesions and less tendon excursion.^32^ These findings may lead to improved clinical patient outcomes.

This study is not without its limitations. Inherent in a cadaveric biomechanical study design, this investigation does not model healing, and the biomechanical properties presented for each tendon construct are at time zero. Moreover, the biomechanical model has been simplified and does not replicate the behavior experienced in the hand and can be considered the worst-case scenario. A limitation associated with soft tissue suture application is the issue of suture creep, which can be related to suture material and tensioning.^24^^,^^33^^,^^34^ In efforts to control this, all samples were preconditioned before cyclic testing and ramp to failure. Related to soft tissue is the quality of tendons from donors. During the harvesting process, no defects or concerns were noted. However, although the EPL may provide a good model for end-to-end tendon repair/transfer, some transfers are performed in an end-to-side fashion. The current model is not suited to test end-to-side tendon coaptations. Additionally, by maintaining matched pair grouping, this minimized the differences related to donor demographics and tissue variability, allowing differences the addition of CoNextions repair system may have had on performance. Timing of CoNextions application has been found to be relatively quick and does not vary much from newer used compared with more experienced surgeons in our experience. However, the time reported in this study may not be an exact average of every surgeon and may vary depending on surgeon’s skill and experience.

The Pulvertaft weave and CoNextions tendon repair device demonstrated similar performance to traditional Pulvertaft tendon weave. The combination technique is faster than the traditional tendon weave and displayed improved performance compared with CoNextions repair alone. Future studies including a clinical study evaluating the outcomes of this tendon transfer technique and comparing it with Pulvertaft weave would be needed to understand the full difference in patient outcomes that this may offer.

## Conflicts of Interest

No benefits in any form have been received or will be received related directly to this article.
